# The Synthesis of Green Palladium Catalysts Stabilized by Chitosan for Hydrogenation

**DOI:** 10.3390/molecules29194584

**Published:** 2024-09-26

**Authors:** Farida Bukharbayeva, Alima Zharmagambetova, Eldar Talgatov, Assemgul Auyezkhanova, Sandugash Akhmetova, Aigul Jumekeyeva, Akzhol Naizabayev, Alima Kenzheyeva, Denis Danilov

**Affiliations:** 1 Laboratory of Organic Catalysis, D.V. Sokolsky Institute of Fuel, Catalysis, and Electrochemistry, Kunaev Str. 142, Almaty 050010, Kazakhstan; 2 Interdisciplinary Resource Center for Nanotechnology, St. Petersburg State University, Universitetskaya nab. 7/9, 199034 St. Petersburg, Russia

**Keywords:** composite, chitosan, palladium catalysts, montmorillonite, polysaccharide

## Abstract

The proposed paper describes a simple and environmentally friendly method for the synthesis of three-component polymer–inorganic composites, which includes the modification of zinc oxide or montmorillonite (MMT) with chitosan (CS), followed by the immobilization of palladium on the resulting two-component composites. The structures and properties of the obtained composites were characterized by physicochemical methods (IRS, TEM, XPS, SEM, EDX, XRD, BET). Pd–CS species covered the surface of inorganic materials through two different mechanisms. The interaction of chitosan polyelectrolyte with zinc oxide led to the deprotonation of its amino groups and deposition on the surface of ZnO. The immobilization of Pd on CS/ZnO occurred by the hydrolysis of [PdCl_4_]^2−^, followed by forming PdO particles by interacting with amino groups of chitosan. In the case of CS/MMT, protonated amino groups of CS interacted with negative sites of MMT, forming a positively charged CS/MMT composite. Furthermore, [PdCl_4_]^2−^ interacted with the –NH^3+^ sites of CS/MMT through electrostatic force. According to TEM studies of 1%Pd–CS/ZnO, the presence of Pd nanoclusters composed of smaller Pd nanoparticles of 3–4 nm in size were observed on different sites of CS/ZnO. For 1%Pd–CS/MMT, Pd nanoparticles with sizes of 2 nm were evenly distributed on the support surface. The prepared three-component CS–inorganic composites were tested through the hydrogenation of 2-propen-1-ol and acetylene compounds (phenylacetylene, 2-hexyn-1-ol) under mild conditions (T—40 °C, P_H2_—1 atm). It was shown that the efficiency of 1%Pd–CS/MMT is higher than that of 1%Pd–CS/ZnO, which can be explained by the formation of smaller Pd particles that are evenly distributed on the support surface. The mechanism of 2-hexyn-1-ol hydrogenation over an optimal 1%Pd–CS/MMT catalyst was proposed.

## 1. Introduction

In recent years, there has been great interest in the preparation and application of organic–inorganic composite materials [1,2,3,4,5,6]. In the design of such materials, the combination of the advantages of both components determines the specific properties of nanocomposites and promotes the formation of composite compounds with desired properties, which positively affect the physicochemical properties of nanocomposite materials. 

A special class of composites are materials obtained by combining polymers and inorganic materials. The modification of the surface of inorganic materials with a polymer layer is widely used to improve their properties [1,2,3,4]. Organo–inorganic structures are also of interest from the position of expanding the possibilities of the application of inorganic materials (metals, oxides, sorbents, and minerals) in various spheres of science and technology [7,8,9,10]. There are two main approaches to surface functionalization with polymers: physical and chemical [1]. The chemical approach is preferable because it excludes polymer desorption due to the chemical bonds between polymer chains and the support surface [1]. 

Due to the requirements of green chemistry, researchers in this field are currently focusing on the use of renewable resources to create new environmentally friendly nanomaterials for wide applications [11,12,13,14,15,16,17,18,19,20,21,22,23]. Polysaccharides (PS) obtained from natural sources are a suitable alternative to synthetic polymers produced from petroleum products [11,12,13,14]. PS have a large number of functional groups in their structure and therefore can create composites with mineral supports and transition metal ions [11,12,13,15,16,18]. Thus, chitosan-based composites have received great attention due to the specific characteristics of chitosan (CS), such as chemical properties, non-toxicity, renewability, and high availability [20,21,22,23]. Research on the use of chitosan in the synthesis of catalysts as a complexing and stabilizing agent has been conducted [20,21]. Due to the presence of active amino groups, thermal stability, and insolubility in organic solvents, chitosan has great promise as a universal carrier for supporting nanoparticles of metal ions for use in various catalytic processes [22,23,24,25,26,27]. The synthesis of chitosan-based copper catalysts, which involves mixing a suspension of chitosan with a metal precursor solution, was proposed in [25]. The developed catalysts (CS@CuSO_4_, CS@Cu(OAc)_2_, CS@Cu_2_O) were investigated in C-O and C-N conjugation reactions without ligands. CS@Cu_2_O was synthesized by the ultrasonic stirring of Cu_2_O and chitosan nanoparticles in toluene. This catalyst demonstrated high activity and stability. Reddy [26] developed palladium catalysts deposited on chitosan (Pd/Chit) by physically mixing chitosan, palladium (II) chloride, sodium hydroxide, and ascorbic acid. The synthesized palladium catalyst showed high reactivity in the reduction reaction of *p*-nitrophenol to *p*-aminophenol in an aqueous medium at 300 K. The TOF was 8.498 h^−1^ and the product yield was 96%. The catalyst showed high stability, and no significant decrease in catalytic activity was observed when used ten times. In [27], Pd catalysts deposited on chitosan microspheres were developed using the electrospraying of a PdCl_2_/chitosan mixture in an aqueous solution of trifluoroacetic acid into an aqueous solution of sodium hydroxide. Next, the chitosan microspheres were cross-linked with glutaraldehyde. The synthesized Pd composite was tested in the Mizoroki–Heck reaction of aromatic iodides with olefinic compounds. At 110 °C, the yields of the products were 82–98%. The good reactivity of the Pd composites deposited on chitosan microspheres is due to the high dispersion of the metal and the small size of the chitosan microspheres. 

The use of chitosan in design catalysts (as support or for stabilization) promotes the formation of composite systems with regulated catalytic properties due to the effect of the polymer on the physicochemical properties (size, shape, morphology) of active metal particles. On the other hand, using chitosan as a catalyst support can provide a number of disadvantages, such as insufficient resistance to abrasion and a low specific surface area. To solve this problem, it is possible to use inorganic sorbents such as metal oxides and aluminosilicates modified by CS as a support material [28,29,30,31]. Montmorillonite (MMT) is a typical layered silicate, whose parallel layers are interconnected by weak electrostatic forces [17]. This structure allows the preparation of different types of polymer-layered silicate composites, depending on the synthesis conditions and the nature of the components [32]. In addition, MMT, due to its large surface area, is a promising material for the synthesis of heterogeneous catalysts based on chitosan [33]. However, to our knowledge, the composites based on CS and MMT have not been practically used in the design of supported metal catalysts.

In our prior works, PS-containing Pd/ZnO catalysts obtained by the green one-pot technique were shown to be promising for use in the hydrogenation of various unsaturated compounds [34,35,36]. In this work, a detailed study of each stage of the formation of three-component polysaccharide-inorganic composites containing metal (Pd), polymer (chitosan), and inorganic sorbents (clay mineral and ZnO) has been carried out for the first time. The obtained results were confirmed by a complex of physicochemical methods of analysis. The reaction ability of the synthesized three-component chitosan–inorganic palladium composites was studied in the low-temperature hydrogenation of 2-propen-1-ol and acetylene compounds (phenylacetylene and 2-hexyn-1-ol). The mechanism of reaction for the hydrogenation of 2-hexyn-1-ol over an optimal 1%Pd–CS/MMT catalyst was also discussed.

## 2. Results

### 2.1. Modification of Inorganic Materials with Chitosan 

The first stage of the modification of inorganic materials (ZnO, MMT) was carried out by the dropwise addition of chitosan (CS) solution to an aqueous suspension of zinc oxide or MMT. The amount of CS adsorbed was assessed by measuring the viscosity of a mother liquor taken after the sorption process and following the determination of the polymer concentration using a calibration curve (Table 1). 

The results obtained shows that ZnO possesses an excellent ability to adsorb chitosan macromolecules. The adsorption degree achieved 97–99% regardless of the amount of CS introduced. In the case of MMT, the degree of chitosan adsorption decreased from 98% to 92% when the amount of the polymer introduced increased from 31 mg to 53 mg. Depending on the amount of chitosan solution introduced, the calculated CS content was found to be 1.0%wt., 2.0%wt., 3.0%wt., and 5.0%wt. for CS/ZnO and 1.0%wt., 2.0%wt., 3.0%wt., and 4.6%wt. for CS/MMT composites. 

The presence of chitosan in the composites was confirmed by IR spectroscopy. Table 2 shows the IR spectra data for chitosan, chitosanium chloride (salt of chitosan with HCl), ZnO, MMT, CS/ZnO, and CS/MMT. For CS, the main characteristic peaks are found at 3410 and 3160 cm^−1^ (O–H and N–H stretching vibration), 2921 and 2853 cm^−1^ (C–H stretching vibration), 1612 cm^−1^ (C=O stretching vibration), 1564 cm^−1^ (N–H in-plane bending vibration), and 1076 cm^−1^ (absorption frequencies of β-d-pyranoside in chitosan) [24,28,37]. The dissolving of chitosan in a dilute HCl solution leads to the protonation of amino groups with the formation of positively charged water-soluble cationic polyelectrolyte [38], while shifting the vibration band of –NH-groups at 1564 cm^−1^ towards shorter wavenumbers [39,40]. The introduction of an acidified chitosan solution into a zinc oxide suspension results in the formation of a CS/ZnO composite, which is accompanied with the shifting of the vibration band of the –NH-groups of the polymer from 1502 to 1559 cm^−1^. This shift can be explained by the deprotonation of amino groups (–NH^3+^) of water-soluble chitosan polyelectrolytes due to their interaction with ZnO. The polymer loses its charge and becomes insoluble.

2R-NH^3+^ + ZnO = 2R-NH_2_↓ + Zn^2+^ + H_2_O

The vibration bands of the –NH-, –OH-, and C–O–C groups of chitosan in the CS/ZnO composite at 1559, 3491, and 1055 cm^−1^ were also shifted to compare with those of the initial chitosan, probably due to the intermolecular interaction of CS with ZnO through van der Waals forces [41]. 

In the case of the CS/MMT composite, a shift in the absorption band of the stretching vibrations of –NH-groups from 1502 to 1512 cm^−1^ was observed, which can be explained by the electrostatic interaction between the protonated amino group of the polymer and the negatively charged sites of the aluminosilicate surface [32]. In addition, the chitosan macrocation can be retained on the sorbent surface due to the interaction of the hydroxyl groups of MMT with the -OH groups of the biopolymer [17,32], as evidenced by the shift of the absorption bands in the 3000–3700 cm^−1^ area, as well as the slight shift of the ν Al–O and ν Si–O bands (Table 2) [17].

**Figure 1:**
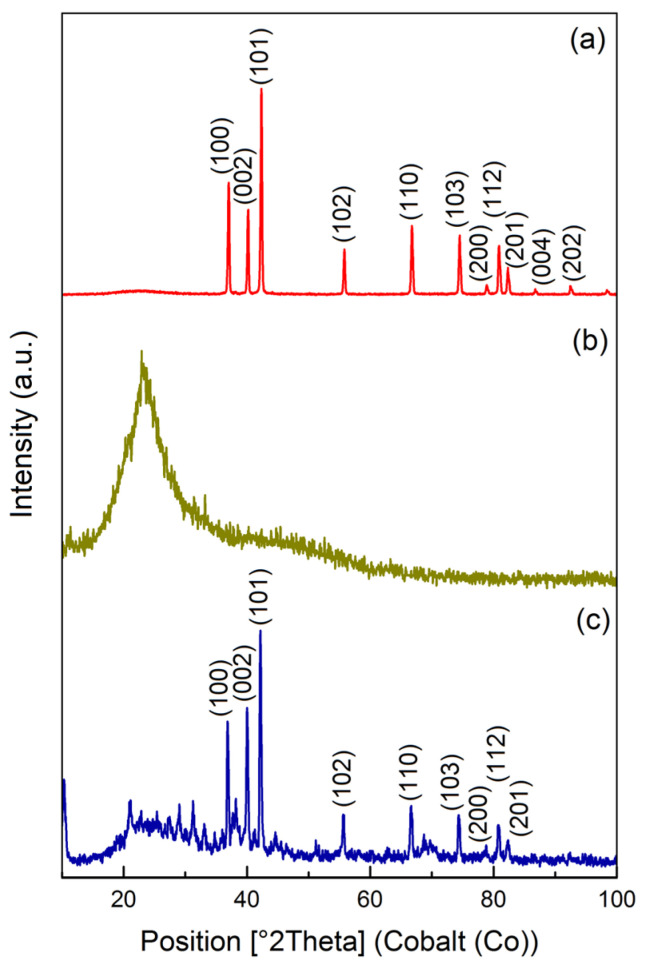
Diffractograms of zinc oxide (**a**), chitosan (**b**), and the 5% CS/ZnO (**c**) composite.

Additional studies on the composite’s structure were carried out by X-ray diffraction (XRD). Figure 1 shows the XRD patterns of zinc oxide, chitosan, and the composite based on them. The characteristic peaks at 37.0°, 40.2°, 42.3°,55.8°, 66.7°, 74.5°, 78.9°, 80.9°, 82.3° observed in both ZnO and CS/ZnO correspond to the (100), (002), (101), (102), (110), (103), (200), (112), and (201) planes of the ZnO wurtzite structure (JCPDS card no. 79-0206) [42]. A broad peak at 20–30° observed in the XRD pattern of CS/ZnO can be attributed to the amorphous phase of the chitosan (Figure 1b). Thus, as expected, the deposition of polysaccharide on the surface of zinc oxide does not lead to a change in its phase state.

According to the literature [43,44], three types of composites—microcomposites and intercalated and exfoliated nanocomposites—can be formed when the polymer is introduced into the layered silicate. The formation of each of the three types of composites is accompanied by a preservation or change in the position (001) of the basal reflection. 

**Figure 2:**
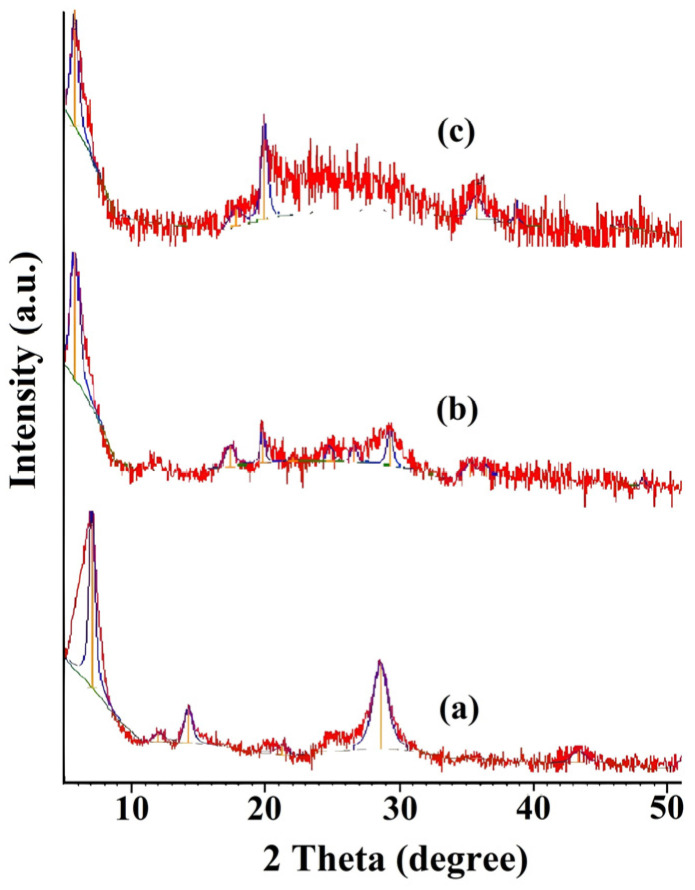
Diffractograms of MMT (**a**), 2% CS/MMT (**b**), and 4.6% CS/MMT (**c**) composites.

The diffractogram of the original montmorillonite sample (Figure 2a) is represented by a wide reflex (001) at 2θ = 7.06° and a second-order reflex at 14.20°, which both indicate that the clay is a sodium montmorillonite with one layer of water molecules in the interlayer space of the crystallites (Na-MMT—12.5 Å). An additional second-order reflex at 2θ = 12.00° (d001 = 14.73 Å) can be corresponded to the Ca form of montmorillonite (Ca-MMT—14.6 Å) [17]. Thus, the clay was shown to be composed of both sodium and calcium forms of MMT.

The modification of MMT with chitosan by the adsorption of the polymer from the aqueous solution promoted the aggregation of MMT particles and the formation of a flake-like precipitate, which was followed by a change in the position of the basal (001) reflection (Figure 2). A comparison of the diffractograms of CS/MMT composites with initial MMT showed an increase in d001 from 12.5 Å to 15.2–15.4 Å. This is probably due to the intercalation of one layer of chitosan polymer chains, 4.4 Å thick [45], into the interlayer space of MMT. In addition, in the XRD pattern of 4.6%CS/MMT, a broad peak at 20–30° appeared, which can be corresponded to the amorphous phase of the chitosan on the MMT surface. 

The textural characteristics and morphology of CS–inorganic composites was studied using low-temperature nitrogen adsorption–desorption and scanning electron microscopy (SEM) methods.

The results of the measurement of the specific surface area of ZnO, MMT, and their CS-modified composites are presented in Table 3.

The results obtained (Table 3) show that the modification of inorganic materials results in the formation of CS/ZnO and CS/MMT composites with decreased surface areas. For example, the specific surface area of ZnO (12.3 m^2^/g) and MMT (100.7 m^2^/g) decreased to 10.2 m^2^/g and 96.1 m^2^/g, respectively, after loading 2% of chitosan on their surface. Moreover, further increases in the amount of polymer being loaded led to a decrease in the specific surface area of the resulting CS/ZnO and CS/MMT composites of up to 7.8 m^2^/g and 86.5 m^2^/g, respectively. Such changes in the specific surface area of inorganic materials can be explained by their surface being blocked with chitosan and the percentage of the blocked surface increasing as the polymer loading increases. 

**Figure 3:**
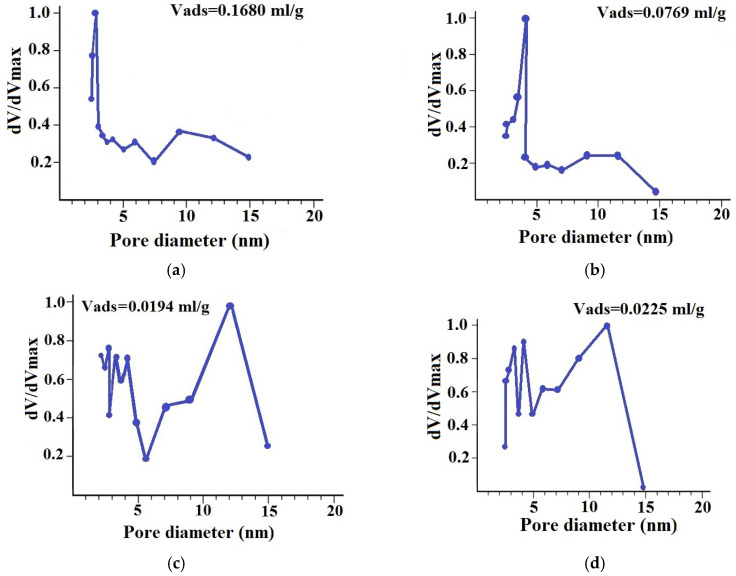
Pore size distributions of MMT (**a**), 2.0%CS/MMT (**b**), ZnO (**c**), and 2.0%CS/ZnO (**d**).

The comparison of the pore size distribution curves also indicates the interaction of chitosan with the surface of the inorganic materials (Figure 3).

The modification of MMT with chitosan causes a redistribution of pore sizes (Figure 3a,b), promoting an increase in the average diameter of mesopores from 2.6 to 3.8 nm and blocking pores with sizes of 4–6 nm, which contributes to a more than twofold decrease in the total pore volume. In the case of coating ZnO with chitosan (Figure 3d), the changes in pore size distribution were less pronounced, which is probably due to the weak interaction of the polymer with the metal oxide surface.

**Figure 4:**
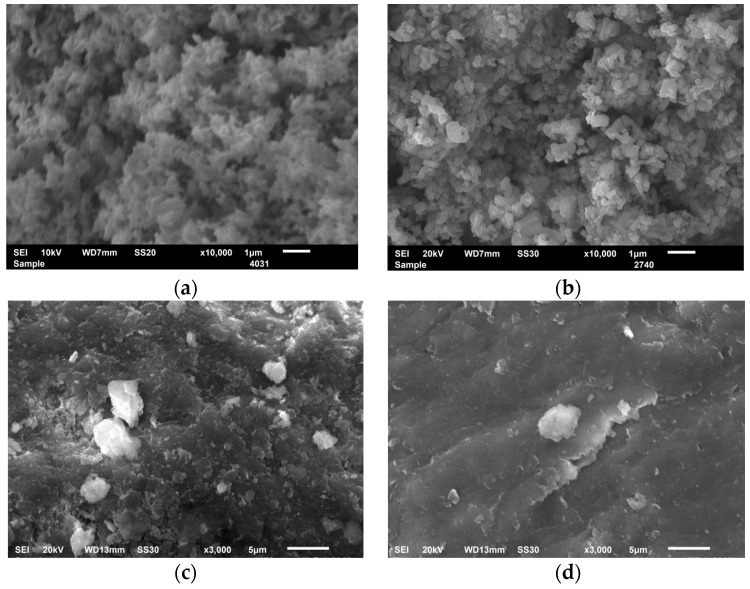
SEM images of zinc oxide (**a**), CS/ZnO (**b**), MMT (**c**) and CS/MMT (**d**).

According to the data of scanning electron microscopy, the initial zinc oxide is a finely dispersed powder (Figure 4a). The modification of zinc oxide with chitosan promotes changes in the particle shape and size, and probably causes them to clump together into larger aggregates due being enveloped by the polymer (Figure 4b).

In the case of the clay mineral, the change in morphology after its modification with chitosan was also observed. SEM images of MMT show thin curved sheets connecting along the basic planes, which leads to the formation of microaggregates. The border between microaggregates is not well traced, and one microaggregate grades gradually into the next (Figure 4c). The surface of the CS/MMT composite is more homogeneous, representing sheets and microaggregates of MMT superimposed on each other, which are covered with a CS layer.

Thus, based on the results obtained, it can be assumed that chitosan, due to the interactions of its –NH- and –OH functional groups with hydroxyl groups of zinc oxide and MMT, forms a macromolecular layer on the surface of inorganic sorbents, changing their surface morphology and textural properties. Chitosan can also change the structure of MMT by intercalating into the interlayer space of the clay mineral (Figure 9a). 

### 2.2. Synthesis of Chitosan–Metal Complexes Supported on Zinc Oxide and MMT

The next stage for the preparation of polymer-modified Pd catalysts is an immobilization of palladium on a polymer–inorganic support. Immobilization was carried out by the adsorption of [PdCl_4_]^2−^ ions on CS/ZnO and CS/MMT composites. The amount of Pd immobilized on a support material was determined by the change in the concentration of [PdCl_4_^2−^] ions in the mother liquor before and after sorption using the photoelectric colorimetric method (PEC).

According to PEC data, 98–99% of the amount of palladium ions introduced was adsorbed on CS/ZnO composites. Thus, the calculated content of Pd in all synthesized Pd–CS/ZnO catalysts was 1 wt.%, which is confirmed by EDX elemental analysis data (Table 4).

A somewhat different situation is observed for palladium sorption on CS/MMT. MMT is a layered silicate with a negatively charged surface and is unable to adsorb the negatively charged palladium ion ([PdCl_4_]^2−^). The modification of MMT with chitosan promotes a change in its surface charge, due to which chitosan-modified composites are able to adsorb anions. So, the adsorption of palladium ([PdCl_4_]^2−^) reaches 86% already at the 1% loading of MMT with chitosan. A further increase in the chitosan content up to 2–5% increases the adsorption of palladium up to 95–99%. These results were confirmed by EDX elemental analysis, according to which the palladium content in Pd–CS/MMT was found to be 0.9–1.2 wt.% (Table 5).

**Figure 5:**
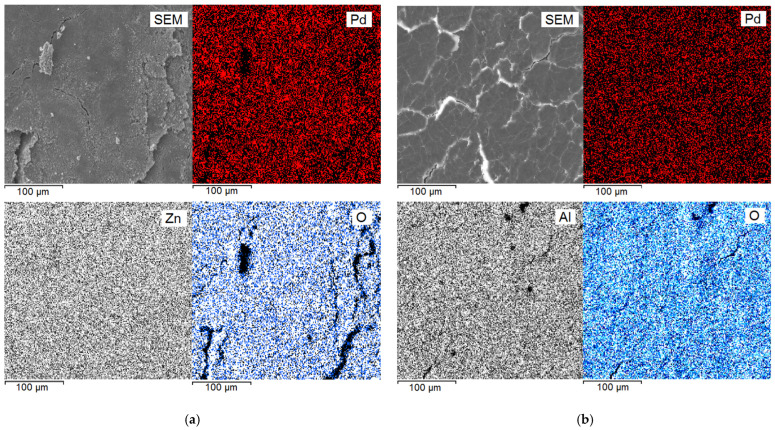
SEM/EDX elemental mapping images of 1%Pd–CS/ZnO (**a**) and 1%Pd–CS/MMT (**b**) catalysts.

Figure 5 shows the SEM and EDX elemental mapping images of Pd, Zn, Al, and O from the 1%Pd–CS/ZnO and 1%Pd–CS/MMT catalysts. All elements (Pd, Zn, O for 1%Pd–CS/ZnO and Pd, Al, O for 1%Pd–CS/MMT) are homogeneously distributed, corresponding to the SEM images, suggesting that Pd is homogeneously immobilized on CS/ZnO and CS/MMT supports.

**Figure 6:**
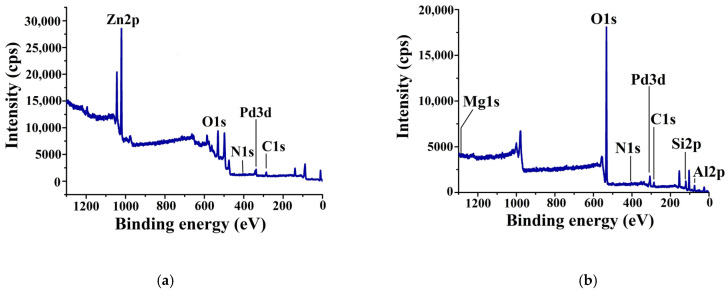
Survey XPS spectrum of 1%Pd-CS/ZnO (**a**) and 1%Pd-CS/MMT (**b**) catalysts.

The presence of the both chitosan and palladium on the surface of the inorganic support was also confirmed by an XPS study. The lines of carbon, nitrogen, and palladium were observed in the survey XPS spectrum of the Pd–CS/ZnO and Pd–CS/MMT catalysts (Figure 6). 

**Figure 7:**
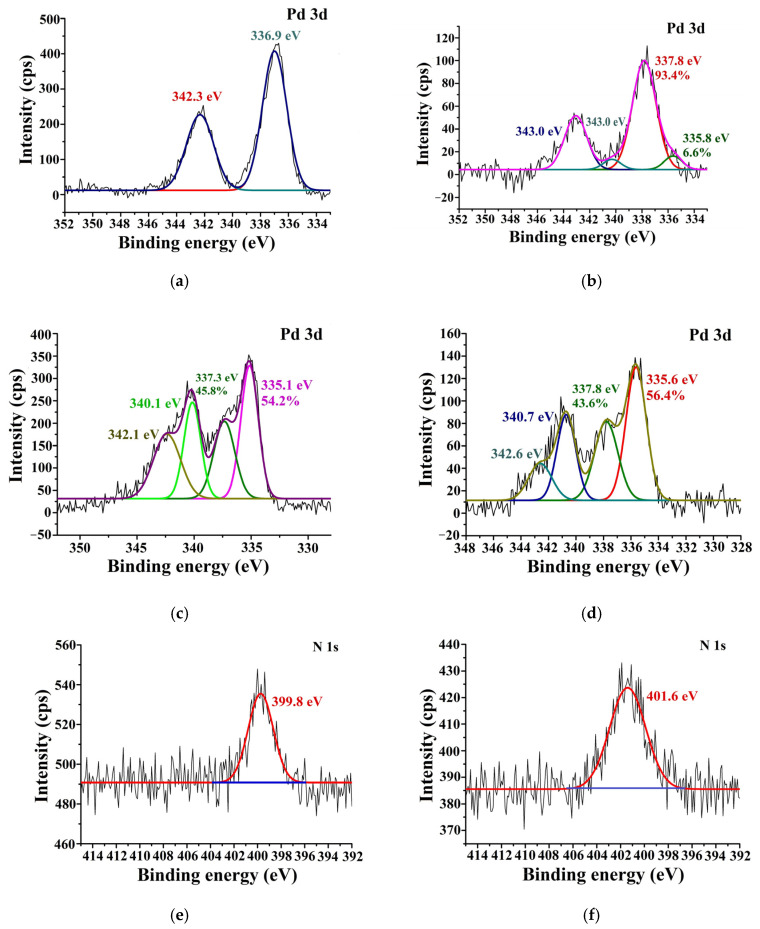
The Pd3*d* (**a**–**d**) and N*1s* (**e**,**f**) XPS spectra of the 1%Pd–CS/ZnO (**a**,**c**,**e**) and 1%Pd–CS/MMT (**b**,**d**,**f**) catalysts before (**a**,**b**) and after (**c**–**f**) treatment with H_2_.

According to the XPS data, palladium in Pd–CS/ZnO occurred in an oxidized state with a 3d_5/2_ binding energy of ~336.9 eV (Figure 7a), which could probably be attributed to the PdO (337.3 eV) [46] formed due to the hydrolysis of [PdCl_4_]^2−^ in the presence of the basic CS/ZnO composite. It is likely that a shift toward smaller energies was caused by the interaction of palladium with the amino group of chitosan [47]. In the spectrum of Pd catalysts supported on CS/MMT (Figure 7b), peaks characteristic of both the oxidized (337.8 eV) and reduced (335.6 eV) forms of palladium were detected. A major peak at 337.8 eV can be attributed to [PdCl_4_]^2−^ [48], while a minor peak at 335.6 eV is corresponding to palladium in a zerovalent state [46]. 

After the treatment of the catalysts with hydrogen in a reactor at 40 °C, most palladium (>50%) was reduced to a zerovalent state. In the Pd3d5/2 XPS spectrum of 1%Pd–CS/ZnO, a new peak at 335.1 eV corresponding to Pd^0^ appeared, which had a negative shift of 0.3 eV [46]. At the same time, the N signal of 1%Pd–CS/ZnO (Figure 7e) had a positive shift of 0.6 eV [47]. On the contrary, in the Pd3d5/2 XPS spectrum of 1%Pd–CS/MMT, a peak of Pd^0^ at 335.6 eV was shifted towards higher energies (0.2 eV) [46], while the N signal of 1%Pd–CS/MMT (Figure 7f) at 401.6 eV could be assigned to the interim state between [PdCl_4_]^2−^-loaded −NH^3+^ (402.1 eV) and free −NH^3+^ (400.6 eV) [48], which was probably caused by the interaction of −NH^3+^ groups of chitosan with both [PdCl_4_]^2−^ and Pd^0^. This suggests that in both cases, the palladium species interact with amino groups of the polymer even after their reduction to a zero-valence state.

**Figure 8:**
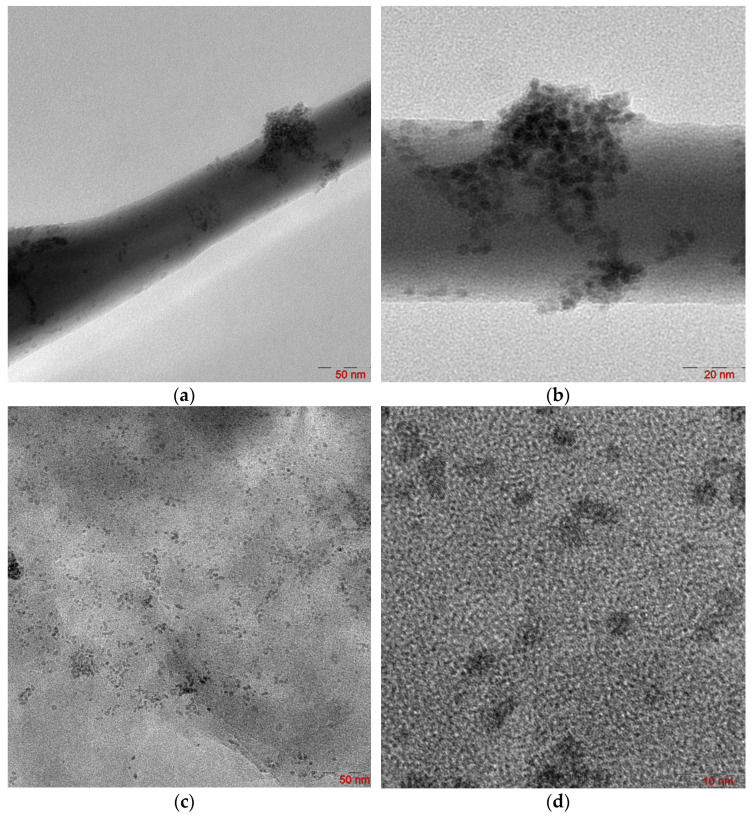
TEM microphotographs of 1%Pd–CS/ZnO (**a**,**b**) and 1%Pd–CS/MMT (**c**,**d**) catalysts at different magnifications.

The interaction between palladium species and amino groups of chitosan can affect the Pd particle size and their distribution on an inorganic support material [35]. Figure 8 shows the transmission electron microscopy (TEM) microphotographs of 1%Pd–CS/ZnO and 1%Pd–CS/MMT catalysts. 

According to TEM studies, the 1%Pd–CS/ZnO catalyst represents separate “islands” of Pd nanoclusters located on different sites of the CS–ZnO support (Figure 8a). The analysis of a TEM microphotograph of the catalyst at a higher magnification level showed that these nanoclusters are composed of smaller spherical Pd nanoparticles of 3–4 nm in size (Figure 8b). In the case of the 1%Pd–CS/MMT catalyst, spherical Pd nanoparticles of 2 nm in size were uniformly distributed on the support surface (Figure 8a,b).

**Figure 9:**
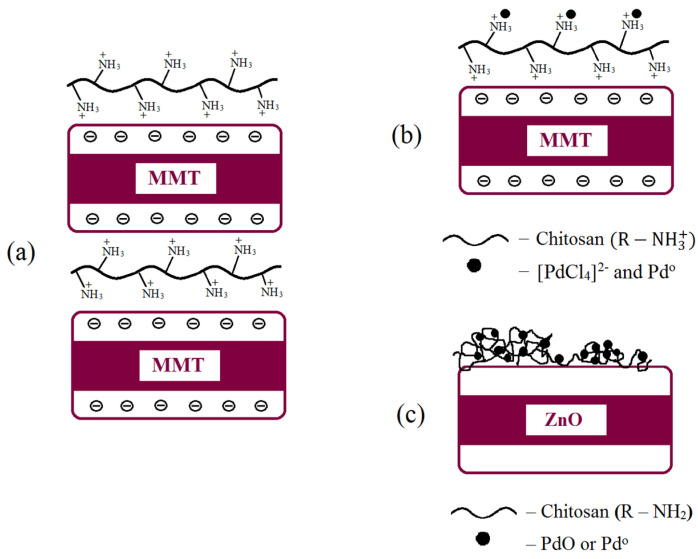
Proposed scheme of the formation of the CS/MMT (**a**), 1%Pd–CS/MMT (**b**), and 1%Pd–CS/ZnO (**c**) composites.

Thus, it was assumed that the basic character of the CS/ZnO composite led to the hydrolysis and quantitative deposition of [PdCl_4_]^2−^ anions on its surface. At the same time, the deposited Pd species can interact with amino groups of chitosan and form nanoclusters on different sites of the CS–ZnO support (Figure 9c). In the case of Pd–CS/MMT, [PdCl_4_]^2−^ anions can interact with –NH^3+^ sites of CS/MMT, forming a supported polymer–metal complex, in which the polymer is an outer-sphere cation (Figure 9b).

### 2.3. Catalytic Properties of Composites

Composites based on polymer-stabilized transition metal nanoparticles show catalytic activity in hydrogenation processes [49,50]. Therefore, the obtained palladium composites were tested in the hydrogenation of 2-propen-1-ol, which is often used as a model compound [51]. 

We showed that the modification of inorganic materials with biopolymers leads to the stabilization and dispersion of the transition metal particles supported on them [35]. Increasing the polysaccharide content in such systems should increase the proportion of stabilized metal particles, but this may create diffusion difficulties for the transferring of substrates to active centers inside the polymer matrix. Therefore, the dependence of the effect of polysaccharide content on the catalytic properties was investigated to determine the optimal composition of the catalysts. The effectiveness of the catalyst was tested under mild conditions (t—40 °C, P—1 atm) in an ethanol medium.

Increasing the polysaccharide content from 1% to 2% in the catalyst composition leads to an increase in the reaction rate (Table 6). As the polymer content of the catalyst is increased up to 5%, the process rate decreases. According to the chromatographic analysis of the reaction products, 100% conversion of the substrate was observed in all cases, while the introduction of the polymer was attributed to an increase in the propanol selectivity of the process. This is probably due to the stabilization of palladium nanoparticles by the polymer, a specific orientational “tuning” of the substrate to the active centers of the catalytic system, preventing isomerization (Table 6).

The testing of the 1% Pd–CS/MMT composite containing 2% polymer in the hydrogenation of 2-propen-1-ol showed that its activity was 1.4 times higher than that of a similar catalytic system in which zinc oxide (1% Pd–CS/ZnO) was used as a support, while the propanol selectivity of both systems had commensurate values and exceeded 80% (Table 7).

The results obtained are probably due to the higher specific surface area of aluminosilicate (100.7 m^2^/g) compared to zinc oxide (12.0 m^2^/g).

The developed 1%Pd–CS/ZnO and 1%Pd–CS/MMT composites containing 2% chitosan were studied in the hydrogenation reaction of acetylene compounds such as phenylacetylene and 2-hexyn-1-ol. 

**Figure 10:**
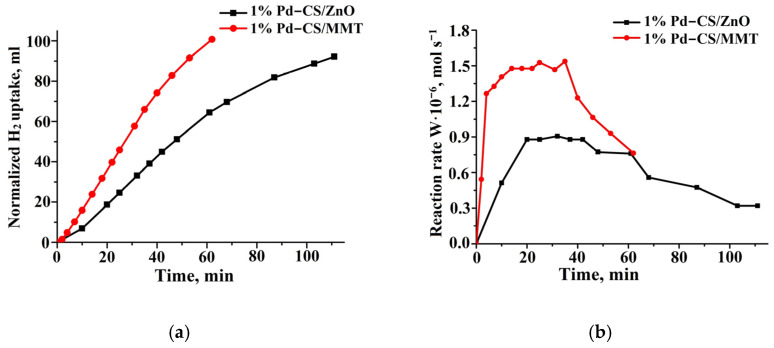
Kinetics of the hydrogen uptake (**a**) and the change in reaction rate (**b**) on 1%Pd−CS/ZnO and 1%Pd−CS/MMT catalysts at the hydrogenation of phenylacetylene. Reaction conditions: T—40 °C, P_H2_—1 atm, m_cat_—0.05 g, solvent C_2_H_5_OH—0.25 mL, and C_sub_—0.09 mol/L.

**Figure 11:**
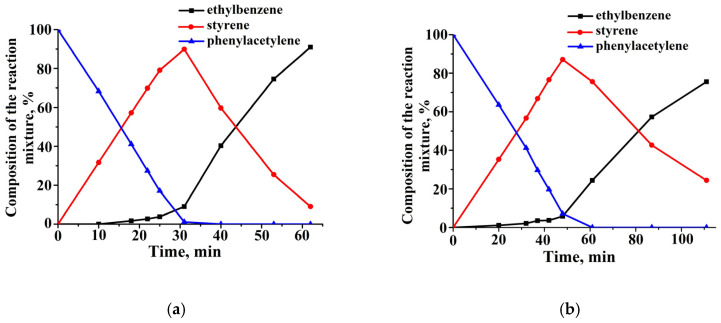
Changes in the composition of the reaction mixture during the hydrogenation of phenylacetylene in the presence of 1%Pd−CS/MMT (**a**) and 1%Pd−CS/ZnO (**b**). Reaction conditions: T = 40 °C, P_H2_—1 atm, m_cat_—0.05 g, solvent C_2_H_5_OH—0.25 mL, and C_sub_—0.09 mol/L.

During phenylacetylene hydrogenation, styrene and ethylbenzene were formed. The hydrogenation rate of phenylacetylene on 1%Pd−CS/MMT was almost two times higher than that on the ZnO-based catalyst and was 1.5 × 10^−6^ mol/s and 0.9 × 10^−6^ mol/s, respectively (Figure 10b). The half-hydrogenation points (50 mL) on these catalysts were reached after 27 and 48 min (Figure 10a). The amount of hydrogen absorbed was in agreement with the chromatographic analysis (Figure 11). The maximum styrene yields were 87.1% on the 1% Pd−CS/ZnO and 89.9% on 1% Pd−CS/MMT. Following the near-total conversion of phenylacetylene to styrene, the latter was hydrogenated to ethylbenzene (Figure 11). 

The comparison of catalytic properties of 1%Pd−CS/ZnO and 1%Pd−CS/MMT are presented in Table 8.

**Figure 12:**
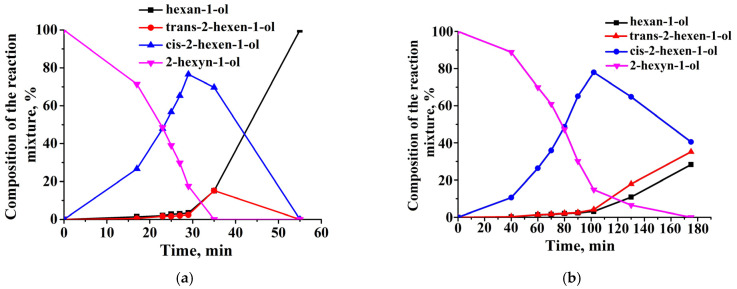
Changes in the composition of the reaction mixture during the hydrogenation of 2-hexyne-1-ol on 1%Pd−CS/MMT (**a**) and 1%Pd−CS/ZnO (**b**). Reaction conditions: T—40 °C, P_H2_—1 atm, m_cat_—0.05 g, solvent C_2_H_5_OH—0.25 mL, and C_sub_—0.09 mol/L.

The hydrogenation of 2-hexyn-1-ol lead to the formation of cis-2-hexen-1-ol, trans-2-hexen-1-ol, and hexan-1-ol, which was confirmed by the chromatographic analysis of the reaction products (Figure 12). In the presence of CS-modified Pd catalysts, supported on MMT, the maximum yield of cis-2-hexen-1-ol was observed at 29 min and was 76.6%, which, with a conversion of 82.4% of 2-hexyn-1-ol, corresponds to the selectivity of 92.9% for cis-2-hexen-1-ol (Figure 12a).

The maximum yield of cis-2-hexen-1-ol on the 1%Pd−CS/ZnO composite was observed at 102 min and was 78% (Figure 12b). The selectivity for 2-hexen-1-ol was 91.5%. The results of 2-hexyn-1-ol hydrogenation on the synthesized CS-containing palladium composites are presented in Table 9.

**Figure 13:**
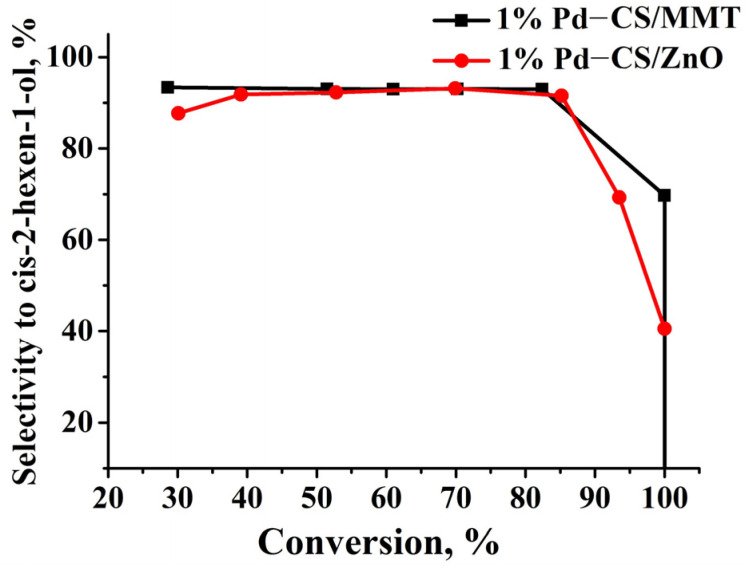
Dependence of the conversion of 2-hexyn-1-ol on selectivity to cis-2-hexen-1-ol in the presence of 1%Pd−CS/MMT and 1%Pd−CS/ZnO composites.

A comparison of the dependence of selectivity on cis-2-hexen-1-ol vs. the conversion rates for 1%Pd−CS/MMT and 1%Pd−CS/ZnO composites is shown in Figure 13. In the presence of 1%Pd−CS/ZnO, the olefin selectivity decreased slightly to 40% when the 2-hexyn-1-ol conversion reached 100%. On 1%Pd−CS/MMT, the process selectivity decreased to 10% when 100% substrate conversion was reached.

Thus, it is shown that the efficiency of the palladium catalyst supported on CS/MMT is higher than that on zinc oxide, which can be explained by the formation of finer palladium particles (2 nm compared to 3–4 nm on ZnO) evenly distributed on the support surface.

**Figure 14:**
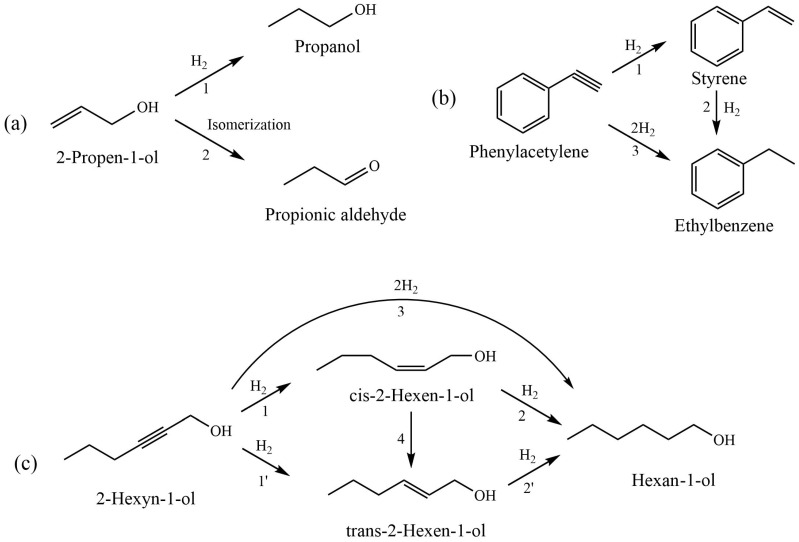
Plausible pathways of the hydrogenation of 2-propen-1-ol (**a**), phenylacetylene (**b**), and 2-hexyn-1-ol (**c**).

Figure 14 shows the plausible reaction pathways for the hydrogenation of 2-propen-1-ol, phenylacetylene, and 2-hexyn-1-ol. The process of the hydrogenation of 2-propen-1-ol to propanol (Figure 14a, reaction 1) is followed by a side reaction of isomerization and the formation of propionic aldehyde (Figure 14a, reaction 2). The hydrogenation of the phenylacetylene is a consecutive process, wherein styrene is an intermediate product and ethylbenzene is formed via the hydrogenation of the C=C bond in the alkene molecule (Figure 14b, reactions 1 and 2). The direct formation of ethylbenzene is also possible (Figure 14b, reaction 3). During the hydrogenation of 2-hexyn-1-ol, the triple C-C bond of the substrate is reduced to double C-C, forming cis-2-hexen-1-ol (Figure 14c, reaction 1), which was then hydrogenated to hexan-1-ol (Figure 14c, reaction 2). At the same time, the accumulation of cis-2-hexen-1-ol is accompanied with the formation of trans-2-hexen-1-ol and hexan-1-ol as side products (Figure 14c, reactions 1′, 4 and 3). It should be noted that the formation of trans-isomers of olefinic alcohol can be carried out by both hydrogenation (Figure 14c, reaction 1′) and isomerization (Figure 14c, reaction 4) reactions. It is well known that catalytic hydrogenations commonly involve the formation of metal hydrides as key intermediates [52,53], which subsequently interact with unsaturated C–C bonds. This suggests that the steric hydrogenation of the triple C-C bond of 2-hexyn-1-ol should result in the formation of cis-2-hexen-1-ol. Consequently, in the initial period of the reaction, the formation of trans-2-hexen-1-ol through the isomerization process (Figure 14c, reaction 4) is more possible. This assumption is supported by chromatography analysis data (Figure 12a), according to which after passing the semi-hydrogenation point, a part of cis-2-hexen-1-ol that had accumulated was transformed to its trans-isomer, which eventually was reduced to hexanol (Figure 14c, reaction 2′). Thus, the semi-hydrogenation of 2-hexyn-1-ol is accompanied with both isomerization and over-hydrogenation processes as side reactions. In more details, in the first step of the reaction, Pd nanoparticles interact with parahydrogen to form PdH_2_ species [53]. Then, PdH_2_ intermediate interacts with the triple C-C bond of 2-hexyn-1-ol to form cis-2-hexen-1-ol, followed by its desorption from active sites of the catalyst. A small amount of cis-2-hexen-1-ol before its desorption from the active sites of the catalyst is transformed to trans-2-hexen-1-ol and hexan-1-ol through isomerization and over-hydrogenation reactions, respectively. 

**Figure 15:**
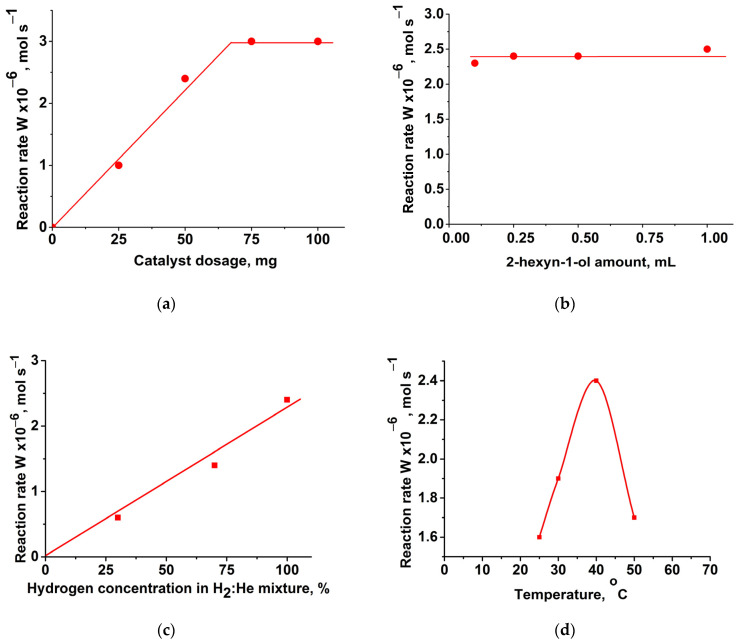
Effect of the variation of reaction parameters on the activity of the 1%Pd-CS/MMT catalyst in the 2-hexyn-1ol hydrogenation: catalyst dosage (**a**); 2-hexyn-1-ol amount (**b**); concentration of hydrogen in hydrogen–helium mixture (**c**); temperature (**d**). Reaction conditions: 40 °C, 0.1 MPa, 100% H_2_, catalyst—25–100 mg, 2-hexyn-1-ol—0.25 mL, ethanol—25 mL (**a**); 40 °C, 0.1 MPa, 100% H_2_, catalyst—50 mg, 2-hexyn-1ol—0.10–1.00 mL, ethanol—25 mL (**b**); hydrogen percentage in H_2_:He gas mixture—30–100 vol%, catalyst—50 mg, 2-hexyn-1ol—0.25 mL, ethanol—25 mL (**c**); 25–40 °C, 0.1 MPa, 100% H_2_, catalyst—50 mg, 2-hexyn-1ol—0.25 mL, ethanol—25 mL (**d**).

To confirm the proposed mechanism of the reaction, a series experiment investigating the kinetics of 2-hexyn-1-ol hydrogenation over the most active 1%Pd-CS/MMT catalyst was performed. The reaction parameters, such as catalyst dosage (25–100 mg), 2-hexyn-1-ol amount (0.1–1.00 mL), hydrogen concentration in the H_2_:He gas mixture (30–100 vol%), and temperature (25–50 °C), were varied (Figure 15).

Figure 15a shows that the W_C≡C_ reaction rate is proportional to the amount of the catalyst in the range of 25–75 mg. Further increases in the catalyst amount (100 mg) did not affect the rate of reaction. This suggests that measurements under the experimental conditions studied (50 mg) are within the kinetic regime. A variation in the substrate amount, in the range of 0.10–1.00 mL (0.9–9.1 mmol), did not significantly affect the rate (Figure 15b), and the reaction to 2-hexyn-1ol seemed to be of zero-order under the reaction conditions studied. The hydrogenation rate decreased linearly with decreasing H_2_ concentrations in the direction of the origin of the coordinates, suggesting that the reaction order to hydrogen is equal to 1 (Figure 15c). A variation in the reaction temperature showed that increasing the temperature from 25 °C to 40 °C led to an increase in the reaction rate (Figure 15d). However, further increases in the temperature, up to 50 °C, resulted in a significant decrease in the hydrogenation rate, which can be explained by collapsing (shrinking) the polymer at higher temperatures [35], making the Pd-active centers less available to the substrate.

Hence, the empirical kinetic equation for hydrogenation of 2-hexyn-1-ol over 1%Pd-CS/MMT is

(1) $$W=\frac{{\mathrm{dH}}_{2}}{\mathrm{dt}}=k_{\mathrm{eff}.}{[H}_{2}][\mathrm{Pd}]$$

The proposed mechanism of the reaction may be presented by the following equations:

(2) $${\mathrm{Pd}+H}_{2}\overset{k_{1}}{\underset{k_{-1}}{⇌}}{\mathrm{PdH}}_{2}$$

(3) $${\mathrm{PdH}}_{2}+C\underset{k_{2}}{\to }\mathrm{PdH}+\mathrm{CH}$$

(4) $$\mathrm{PdH}+\mathrm{CH}\underset{k_{3}}{\to }{\mathrm{Pd}+\mathrm{CH}}_{2}$$

(5) $$\mathrm{Pd}+\mathrm{CH}\underset{k_{4}}{\to }\mathrm{Pd}+\mathrm{CH}\prime$$

where C is 2-hexyn-1-ol; CH is cis-2-hexen-1-ol; CH′ is trans-2-hexen-1-ol; and CH_2_ is hexan-1-ol.

According to chromatography analysis data (Figure 12a), in the initial period of the reaction (before achieving the semi-hydrogenation point), the accumulation of cis-2-hexen-1-ol occurred much faster than the formation of trans-2-hexen-1-ol and hexan-1-ol. Consequently, in the first approximation, the steady-state reaction rate can be expressed as follows (based on Equations (2) and (3)):

(6) $$-\frac{{\mathrm{dH}}_{2}}{\mathrm{dt}}=\frac{k_{1}k_{2}[\mathrm{Pd}][H_{2}][C]}{k_{-1}+k_{2}[C]}$$

When the reaction (3) is fast, i.e., *k*_2_[C] >> *k*_−1_, then the obtained Equation (6) conforms to the empirical one (1). Thus, *k*_eff._ = *k*_1_.

Taking into account the hydrogen solubility in ethanol [54], the rate constant of the second order is 82.9 L/mol·s at 40 °C and P_H_ = 0.1 MPa.

The dependence of the rate constant on the temperature in Arrhenius coordinates is linear. E_act._ = 15.6 kJ/mol (H^#^ = E_act._ − RT = 13.0 kJ/mol; S^#^ = −0.167 kJ/mol·K). The high negative entropy of activation suggests the easy formation of PdH_2_ intermediates, which, according to the proposed mechanism, is a limiting step of the reaction. 

## 3. Materials and Methods

### 3.1. Materials

Chitosan (CS) is a polysaccharide whose macromolecules consist of randomly bonded β-(1-4) d-glucosamine links and *N*-acetyl-d-glucosamine [27,28]. The degree of deacetylation is 85%, Mw = 250,000 (Sigma-Aldrich, St. Louis, MO, USA). Palladium (II) chloride (59–60% Pd) and zinc oxide (chemically pure) were acquired from Sigma-Aldrich, St. Louis, MO, USA. Montmorillonite (MMT) from Tagansky deposit (Ust-Kamenogorsk, Kazakhstan) produced by LLP ‘‘Sorbent’’ (Ust-Kamenogorsk, Kazakhstan).

2-Propen-1-ol, phenylacetylene, and 2-hexyn-1-ol were acquired from Sigma-Aldrich, St. Louis, MO, USA. Ethanol (reagent) was purchased from Talgar Alcohol LLP (Talgar, Kazakhstan) and purified via distillation.

### 3.2. Preparation of Composites

The synthesis of chitosan–inorganic composites is based on the adsorption of polysaccharides on the surface of the inorganic material, as well as the adsorption of metal ions onto the polymer-modified composite from the aqueous solution [35,36].

#### 3.2.1. Preparation of CS/MMT(ZnO) Composites

Chitosan (1 g) was dissolved in 100 mL of a 1% hydrochloric acid solution at 60 °C and stirred for 4 h to prepare a 1% chitosan solution. Then, the 1% chitosan solution was added to the aqueous suspension of MMT (ZnO) (1 g in 15 mL of water) at room temperature under constant stirring. Furthermore, the volume of the mixture was brought to 20 mL and stirred for 2 h. The obtained composites were kept in the mother liquor until their complete precipitation, after which the precipitate was washed with water and dried in air.

The polymer content in composites was estimated on an Ubbelohde viscometer by the change in the viscosity of the mother liquor before and after sorption using calibration curves. The amounts of the chitosan solution introduced were taken from the calculation to obtain composites with the polysaccharide contents of 1, 2, 3, and 5 wt. %. 

#### 3.2.2. Preparation of Composites Based on CS–Palladium Complexes Fixed on MMT (ZnO) 

To an aqueous suspension of the CS-containing composites obtained in Section 3.2.1. (1 g in 15 mL of water), 5 mL of an aqueous solution of palladium(II) salt (K_2_PdCl_4_) containing 0.0101 g of Pd was added dropwise at room temperature and stirred for 3 h. The obtained composites were kept in the mother liquor for at least 10 h, after which the precipitate was washed with water and dried in air. The amount of Pd adsorbed was determined by the difference in the metal ion concentrations in the mother liquor before and after sorption. The concentration of palladium ions was determined on the spectrophotometer SF-2000 (OKB Spectr, Saint-Petersburg, Russia). The calibration of the spectrophotometer was carried out using a series of standard solutions at wavelengths λ_Pd_ = 425.

### 3.3. Characterization of the Composites by Physicochemical Methods

IR spectra were obtained using a Nicolet iS5 from Thermo Scientific (Waltham, MA, USA), with a resolution of 3 cm^−1^ in the 4000–400 cm^−1^ region. Pellets for infrared analysis were obtained by grinding a mixture of a 1 mg sample with 100 mg dry KBr, followed by pressing the mixture into a mold.

X-ray diffraction (XRD) patterns from samples based on ZnO were recorded on a DRON-4-0.7 diffractometer from Bourevestnik (Saint Petersburg, Russia), with Co-Kα radiation at a wavelength of 0.179 nm. XRD patterns from MMT-containing samples were obtained on a PANalytical X’Pert MPD PRO diffractometer (PANalytical, Almelo, The Netherlands) in copper-filtered radiation with a wavelength of 0.154 nm. The preparation of MMT-containing samples for analysis was carried out by pipetting an aqueous suspension of the sample onto a glass plate followed by air drying until water was completely removed.

The specific surface area and porosity of the samples were measured by the Brunauer–Emmett–Teller (BET) method using an Accusorb analyzer (Micromeritics, Norcross, GA, USA). The studied samples were pre-degassed in a vacuum at 60 °C for 4 h, and then the adsorption isotherm was taken. The gas adsorbent was nitrogen.

Scanning electron microscopy (SEM) micrographs were obtained on a scanning electron microscope JSM-6610 LV (“JEOL” Ltd., Tokyo, Japan) at an accelerating voltage of 15–20 kV. EDX elemental analysis was performed using an energy-dispersive detector built into the microscope (EDX Oxford Instruments, Oxford, UK).

Transmission electron microscopy (TEM) micrographs were obtained on a Zeiss Libra 200FE transmission electron microscope (Carl Zeiss, Oberkochen, Germany) with an accelerating voltage of 100 kV. 

X-ray photoelectron spectra (XPS) of palladium composites were recorded on an ESCALAB 250Xi X-ray and ultraviolet photoelectron spectrometer (Thermo Fisher Scientific, Waltham, MA, USA). 

### 3.4. Methodology of Hydrogenation

The hydrogenation of the 2-propen-1-ol and acetylene compounds (2-hexyn-1-ol and phenylacetylene) was carried out in a thermostated glass reactor according to the procedure described in Ref [36]. The reaction was carried out in an ethanol medium (25 mL) at atmospheric hydrogen pressure and a temperature of 20–50 °C, under intensive stirring (600–700 oscillations per minute). Before hydrogenation, the nanocatalyst (0.05 g) was reduced with hydrogen in the reactor for 30 min under conditions of intensive stirring. After the hydrogen treatment, a substrate (14.87 mol/L of 2-propen-1-ol and 0.09 mol/L 2-hexyn-1-ol and phenylacetylene) was added to the reactor.

The hydrogenation products were analyzed using gas–liquid chromatography on a Chromos GC1000 chromatograph (Chromos Engineering, Dzerzhinsk, Russia) with a flame ionization detector in isothermal mode. A BP21 capillary column (FFAP) with a polar phase (PEG modified with nitroterephthalate) was used. This device was 50 m in length and 0.32 mm in inner diameter. The column temperature was 90 °C, the injector temperature was 200 °C, and helium served as the carrier gas. A total of 0.2 mL of the sample was investigated. Selectivity was calculated as the fraction of the target product present in the reaction products at a given degree of substrate conversion.

## 4. Conclusions

The present work provides a detailed study of each stage of the formation of three-component 1%Pd−CS/ZnO and 1%Pd−CS/MMT composites. The characterization of two- and three-component composites using physicochemical methods revealed some of the following noticeable facts: (1) Interaction of the water-soluble form of chitosan with zinc oxide lead to the deprotonation of the polymer amino groups and the chitosan macromolecule became insoluble, covering the surface of ZnO. (2) Protonated amino groups of chitosan interacted with negative sites of MMT, forming a positevely charged CS/MMT composite. (3) Chitosan covered the surface of both MMT and ZnO, and the percentage of the surface blocked increased as the polymer loading increased. (4) In the case of CS/MMT, an intercalation of one layer of chitosan polymer chains into the interlayer space of MMT also took place. (5) The quantitative immobilization of palladium on chitosan-modified zinc oxide occurred due to the basic character of the CS/ZnO composite, providing the hydrolysis of [PdCl_4_]^2−^ anions to form PdO. The resulting PdO particles of 3–4 nm in size interacted with amino groups of chitosan, forming larger aggregates (nanoclusters) on the different sites of CS/ZnO. (6) [PdCl_4_]^2−^ anions interacted with –NH^3+^ sites of CS/MMT, forming a supported polymer–metal complex, in which the chitosan was an outer-sphere cation. 

The developed three-component Pd−CS–inorganic composites were tested in the hydrogenation of 2-propen-1-ol and acetylene compounds (phenylacetylene, 2-hexyn-1-ol) under mild conditions (T—40 °C, P_H2_—1 atm). The results show the great potential of the 1%Pd−CS/MMT as a catalyst for the hydrogenation of unsaturated compounds. This composite is characterized by the presence of 2 nm CS-stabilized palladium particles uniformly distributed on the support surface. The investigation of the kinetics of 2-hexyn-1-ol hydrogenation over the most active 1%Pd-CS/MMT catalyst confirmed the proposed mechanism of the reaction: (1) Pd nanoparticles of the catalyst interact with H_2_ to form PdH_2_ intermediate; (2) the PdH_2_ interacts with the triple C-C bond of 2-hexyn-1-ol to form cis-2-hexen-1-ol, which is then desorbed from the active sites of the catalyst; (3) a small amount of cis-2-hexen-1-ol before its desorption from the active sites is transformed to trans-2-hexen-1-ol and hexan-1-ol through isomerization and over-hydrogenation reactions, respectively. The limiting step of the proposed mechanism is the fomation of PdH_2_ species, which, acording to the calculations performed, are formed quite easily (S^#^ = −0.167 kJ/mol·K).

## Figures and Tables

**Table 1:** The results of the assessment of chitosan content in composites.

m(CS) in the Initial Solution, mg	m(CS) in Solution after Sorption, mg	m(CS) Adsorbed, mg	Adsorption Degree, %	CS Content, %
CS/MMT				
10.1	0.4	9.7	96.0	1.0
20.4	0.4	20.0	98.0	2.0
30.9	0.4	30.5	98.7	3.0
52.6	4.2	48.4	92.0	4.6
CS/ZnO				
10.1	0.3	9.8	97.0	1.0
20.4	0.3	20.1	98.5	2.0
30.9	0.3	30.6	99.0	3.0
52.6	0.4	52.2	99.2	5.0

**Table 2:** The results of the IR spectroscopy of the studied samples.

Sample	νOH νNH	νCH	δNH	νC–O–C ν(C–C)_K_ ν(C–O)_K_	νZn–O νSi–O νAl–O
Chitosan	3410 3160	2921 2853	1616 1564	1076 1037	
Chitosanium chloride	3425 3144	2935 2861	1622 1502	1074 1037	
ZnO	3444				494 446
MMT	3630 3430				1030 914 527
CS/ZnO	3569 3491 3226 3142	2925 2862	1621 1559	1153 1055 1031	478 442
CS/MMT	3636 3429 3179	2920 2842	1657 1512	blocked by the MMT signal	1034 920 530

**Table 3:** Specific surface area of zinc oxide, MMT, and CS-modified composites.

Sample	Specific Surface Area, m^2^/g	Blocked Surface, %
ZnO	12.3	-
2.0%CS/ZnO	10.2	17.1
5.0%CS/ZnO	7.8	36.6
MMT	100.7	-
2.0%CS/MMT	96.1	4.6
4.6%CS/MMT	86.5	14.1

**Table 4:** Sorption of palladium on CS-modified ZnO.

PS Content, %	m(Pd) in Initial Solution, mg	m(Pd) in Solution after Sorption, mg	m(Pd) Adsorbed, mg	Ads. Degree, %	Pd Content, %	
					FEC	Elem. Analysis
1	10.1	0.1	10.0	99.0	1.0	0.97
2		0.1	10.0	99.0	1.0	1.30
3		0.1	10.0	99.0	1.0	n.d.*
5		0.1	10.0	99.0	1.0	n.d.

* n.d.—not detected.

**Table 5:** Sorption of palladium on CS-modified MMT.

PS Content, %	m(Pd) in Initial Solution, mg	m(Pd) in Solution after Sorption, mg	m(Pd) Adsorbed, mg	Ads. Degree, %	Pd Content, %	
					FEC	Elem. Analysis
1	10.1	1.4	8.7	86.1	0.9	0.90
2		0.5	9.6	95.0	1.0	0.98
3		0.1	10.0	99.0	1.0	1.10
5		0.1	10.0	99.0	1.0	1.22

**Table 6:** Hydrogenation of 2-propen-1-ol in the presence of 1%Pd–CS/ZnO catalysts containing different amounts of chitosan *.

Chitosan Content,%	Reaction Rate(W)·10^−6^, mol/s	Selectivity, %		Conversion, %
		Propanal	Propanol	
1	3.6	19.6	80.4	100
2	4.3	17.2	82.8	100
3	3.3	17.0	83.0	100
5	2.7	15.6	84.4	100

* Experimental conditions: m_cat_—0.05 g; C_sub_—14.87 mol/L; solvent ethanol—25 mL; T—40 °C; P_H2_—1 atm.

**Table 7:** Catalytic properties of 1% Pd–CS/ZnO and 1% Pd–CS/MMT composites in the hydrogenation of 2-propen-1-ol *.

Support	Reaction Rate (W) · 10^−6^, mol/s	Selectivity, %		Conversion, %
		Propanal	Propanol	
ZnO	4.3	17.2	82.8	100
MMT	6.0	15.2	84.8	100

* Experimental conditions: m_cat_—0.05 g; C_sub_—14.87 mol/L; solvent ethanol—25 mL; T —40 °C; P_H2_—1 atm.

**Table 8:** Results of the hydrogenation of phenylacetylene on 1% Pd−CS/ZnO and 1% Pd−CS/MMT composites *.

Catalyst	W_max_·10^−6^, mol s^−1^	Selectivity, %	Conversion, %
1%Pd−CS/MMT	1.5	90.9	98.9
1%Pd−CS/ZnO	0.9	93.8	92.9

* Experimental conditions: m_cat_—0.05 g; C_sub_—0.09 mol/L; solvent ethanol—25 mL; T—40 °C; P_H2_—1 atm.

**Table 9:** The results of 2-hexyn-1-ol hydrogenation on the synthesized f 1% Pd−CS/ZnO and 1% Pd−CS/MMT composites *.

Catalyst	W·10^−6^, mol s^−1^		Selectivity to cis-2-hexen-1-ol, %	Conversion, %
	C≡C	C=C		
1%Pd−CS/MMT	2.4	2.4	92.9	82.4
1%Pd−CS/ZnO	0.3	0.3	91.5	85.2

* Experimental conditions: m_cat_—0.05 g; C_sub_—0.09 mol/L; solvent ethanol—25 mL; T—40 °C; P_H2_—1 atm.

## Data Availability

The data that support the findings of this study are available from the corresponding author upon reasonable request.
